# Optimization algorithms for functional deimmunization of therapeutic proteins

**DOI:** 10.1186/1471-2105-11-180

**Published:** 2010-04-09

**Authors:** Andrew S Parker, Wei Zheng, Karl E Griswold, Chris Bailey-Kellogg

**Affiliations:** 1 Department of Computer Science, Dartmouth College, Hanover, NH 03755, USA; 2 Thayer School of Engineering, Dartmouth College, Hanover, NH 03755, USA

## Abstract

**Background:**

To develop protein therapeutics from exogenous sources, it is necessary to mitigate the risks of eliciting an anti-biotherapeutic immune response. A key aspect of the response is the recognition and surface display by antigen-presenting cells of epitopes, short peptide fragments derived from the foreign protein. Thus, developing minimal-epitope variants represents a powerful approach to deimmunizing protein therapeutics. Critically, mutations selected to reduce immunogenicity must not interfere with the protein's therapeutic activity.

**Results:**

This paper develops methods to improve the likelihood of simultaneously reducing the anti-biotherapeutic immune response while maintaining therapeutic activity. A dynamic programming approach identifies optimal and near-optimal sets of conservative point mutations to minimize the occurrence of predicted T-cell epitopes in a target protein. In contrast with existing methods, those described here integrate analysis of immunogenicity and stability/activity, are broadly applicable to any protein class, guarantee global optimality, and provide sufficient flexibility for users to limit the total number of mutations and target MHC alleles of interest. The input is simply the primary amino acid sequence of the therapeutic candidate, although crystal structures and protein family sequence alignments may also be input when available. The output is a scored list of sets of point mutations predicted to reduce the protein's immunogenicity while maintaining structure and function. We demonstrate the effectiveness of our approach in a number of case study applications, showing that, in general, our best variants are predicted to be better than those produced by previous deimmunization efforts in terms of either immunogenicity or stability, or both factors.

**Conclusions:**

By developing global optimization algorithms leveraging well-established immunogenicity and stability prediction techniques, we provide the protein engineer with a mechanism for exploring the favorable sequence space near a targeted protein therapeutic. Our mechanism not only helps identify designs more likely to be effective, but also provides insights into the interrelated implications of design choices.

## Background

The majority of all therapeutic proteins elicit an anti-biotherapeutic immune response (aBIR) in human patients receiving treatment [1]. The clinical effects of such a response may include various rapidly manifested anaphylactic responses, a reduction of therapeutic efficacy, and in rare cases cross-reactivity of anti-drug antibodies with endogenous patient proteins resulting in a form of induced autoimmunity [2]. Wide concern over these issues has focused biopharmaceutical researchers on the immunogenicity of protein therapeutics, and has driven the search for strategies to detect, assess, and ameliorate potentially deleterious immune responses [3-5].

While there exists a variety of factors that influence a protein therapeutic's immunogenicity [6,7], we focus here on the effect of a protein's origins. Specifically, non-human proteins exhibit a disproportionately high frequency of immunogenicity in humans as a result of the classical immune response [8]. In contrast, proteins of human origin are more likely to be recognized as "self," or to meet the "criteria of continuity" [9]. The goal is thus to engineer variants of the foreign protein that also are recognized as "self." For therapeutic antibodies, whose structure and function are well understood, immunogenicity reduction may be realized by rational grafting of key functional residues from an exogenous therapeutic antibody onto a human antibody framework [10-14]. The resulting chimeric antibody maintains the binding specificity and affinity of the exogenous therapeutic candidate, but the majority of the protein is comprised of human-derived amino acid sequences, thereby reducing the propensity for aBIR. The prevalence of chimeric and humanized antibodies among FDA approved therapeutics [15] as well as a detailed meta-analysis [16] provide overwhelming evidence for the efficacy of this approach as a whole. However, there remains a considerable empirical, trial-and-error component, even in "rational" approaches [17]. Rational grafting techniques require a precise knowledge of structure-function relationships, as well as a modular structure common to the exogenous therapeutic candidate and a homologous human protein. With the advanced state of knowledge for immunoglobulin proteins, therapeutic antibodies inherently satisfy these prerequisites. However, exogenous enzymes, signaling peptides, and other classes of non-human proteins represent a potentially massive pool of biotherapeutic agents. To effectively tap this reservoir of next generation drugs, more advanced deimmunization strategies are required to address the fact that many of these candidates do not possess common modular structures and frequently have no homologous human counterpart.

One alternative to humanization by rational grafting is the identification and modification of immunogenic peptide fragments of a protein, or T-cell epitopes, that drive the aBIR. These peptides are derived from proteolytic processing of protein that has been internalized by antigen presenting cells (Trombetta and Mellman [18] provide a detailed review). The peptide fragments are bound within the groove of type II major histocompatibility complex proteins (MHC II), which are then transported to the surface of the immune cell where the peptide-MHC II complex is displayed to the extracellular environment. Should the displayed peptides constitute immunogenic sequences, they will form ternary peptide-MHC II-T-cell receptor complexes with surface receptors of cognate white blood cells. The resulting signaling cascade leads to a coordinated immune response against the offending protein. To avoid such a response, it is sometimes possible to identify the most immunogenic peptide fragments of a candidate protein, and to subsequently mutagenize one or more of the corresponding residues so as to disrupt the peptide fragment's capacity to complex with the MHC II and/or T-cell receptors. This process has been successfully applied to numerous therapeutic candidates including staphylokinase [19], factor VIII [20], and a *β*-lactamase [21]. Deimmunization by epitope deletion suffers from the limitation of being exceptionally time and resource intensive. Traditionally, the approach entails synthesizing and testing the immunogenicity of large panels of peptides from the native protein, performing alanine scanning mutagenesis on the most immunogenic fragments to pinpoint critical MHC II binding residues, incorporating deimmunizing mutations into the full length protein, and finally testing the functionality and immunogenicity of the engineered protein variants, only a small fraction of which are likely to retain high activity and/or constitute globally deimmunized candidates. More advanced implementations of this strategy exchange functionally relevant mutations for alanine mutations, but only late in the experimental cycle.

Computational methods have been employed to aid the identification of mutations that can effectively eliminate MHC II binding. Often computational analyses are performed on only a small subpopulation of peptides that have been preselected from a much larger pool of possibilities [22,23]. These approaches also typically focus on a minimal set of only the most immunogenic peptides (typically 1-3 peptides), and therefore cannot be guaranteed to provide globally optimal sequences. Alternatively, numerous computational tools have been developed for immunogenicity prediction for an entire protein, based on its amino acid sequence [24], and the efficacies of several alternative methods have been evaluated in head-to-head comparisons [25,26]. Some such algorithms have been used to identify immunogenic peptides in practical biotherapeutics [27,28]; our goal is to integrate such immunogenicity analyses within optimization algorithms that reduce predicted immunogenicity while accounting for structural and functional consequences.

In order to address the shortcomings of earlier approaches, this paper presents a novel protein design method in which protein sequences are computationally optimized to produce variants that are more likely to exhibit both low inherent immunogenicity and high level functionality. These are two competing concerns - mutations introduced to reduce immunogenicity may produce unstable or inactive proteins. We establish as our primary optimization objective reduction of immunogenicity, according to predicted T-cell epitopes within the sequence [25]. In order to also address the concern of stability/activity, we identify for each residue position those mutations that are deemed acceptable according to sequence and/or structure-based analyses. A dynamic programming approach then finds globally optimal and near-optimal sets of these acceptable mutations that minimize the occurrence of predicted epitopes.

**Figure 1:**
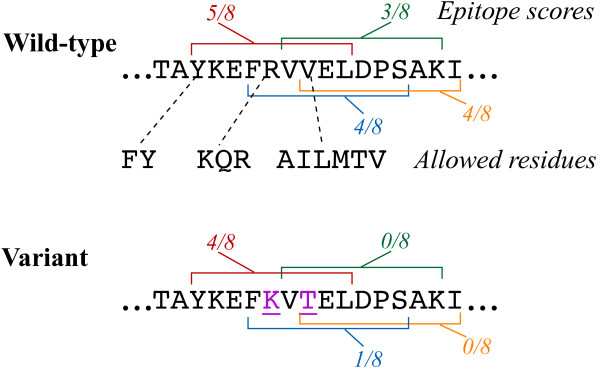
**Deimmunization overview**. We employ T-cell epitope predictors to score each 9-mer peptide for potential immunogenicity. In this example (staphylokinase residues 71-87; see the Results section), four peptides are deemed immunogenic, as they are predicted to be recognized by sever-al of the 8 most representative MHC II alleles. We employ sequence and structure analysis to identify for each position which residues are acceptable; only a few examples are shown. Our algorithms select a specified number of mutations (here two mutations, underlined in the variant) from the acceptable ones, so as to minimize the resulting epitope score. Note that a single substitution at a "promiscuous" amino acid can reduce recognition of multiple overlapping epitopes, and need not be at the so-called "anchor" position.

Our methods provide a number of significant extensions to the state of the art. They are not limited to deimmunization of antibodies (as are simple rational grafting techniques), but can also be applied in engineering immunotolerant versions of more complex proteins, such as therapeutic enzymes. Our approach seamlessly integrates immunogenic peptide identification, mutagenic deimmunization, and functional/structural analysis of potential mutations, employing well-established and effective tools for prediction of epitopes and for evaluation of stability changes. Our dynamic programming-based algorithms are guaranteed to find globally optimal sets of mutations, avoiding the pitfall of making a mutation to mitigate one epitope but inadvertently introducing a new overlapping epitope. We provide the protein engineer with flexibility in setting a desired threshold for immunogenicity, limiting the number of mutations to consider, and in targeting specific MHC alleles. Finally, in contrast to traditional experimental and computational techniques, our methods preferentially guide mutations to the most promiscuous immunogenic amino acids, i.e., those that are elements of two or more overlapping immunogenic peptides (Fig. 1).

We apply our methods to optimize variants of several different protein therapeutics that have previously been targeted for deimmunization by other approaches. We characterize the space of sequences near these targets, identifying variants that are predicted to be less immunogenic than wild-type but still stable, i.e., deleting some predicted epitopes while using only conservative substitutions. We find a number of variants that, in comparison to earlier designs, contain fewer predicted epitopes for a given number of substitutions, or, viewed the other way, use fewer substitutions to delete a similar number of epitopes. Our approach targets many of the same immunogenic regions as identified by experimental studies, even when not specifically focused. Furthermore, by restricting substitutions to be relatively conservative (as assessed under several different models), our variants are likely to maintain greater thermodynamic stability.

## Methods

Our overall goal is to select, from the mutations deemed acceptable, a set that efficiently reduces the occurrence of predicted T-cell epitopes. We now formalize this problem; Fig. 1 illustrates.

**Problem 1 (Deimmunization) ***Given a protein sequence S of length n, determine a variant S' minimizing *, *such that *∀*i*: *S'*[*i*] ∈ *M*(*i*), *where*

• *e *: *A*^9 ^→ [0, 1] *returns the *epitope score *for a peptide (we assume a 9-mer; see below) in the range of 0 to 1, where lower is better*

• *M *: {1, 2, ..., *n*} → 2^*A *^*provides the *allowed residues, *indicating which amino acids (including at least the wild-type) may be considered at each residue position*

Here and throughout, we use *A *= {A,C, ..., Y} for the set of amino acids; sequences are 1-indexed; and the notation *X*_*i*..*j *_indicates the substring of *X *from position *i *to *j*, inclusive.

A number of experimentally-validated bioinformatics tools exist to predict immunogenicity (as encoded in *e*) and changes in stability (*M*). Our current implementation supports several state-of-the-art tools [29,30], but is modular and can readily support others [31-33].

### Immunogenicity evaluation

T-cell epitope predictors encapsulate the underlying specific recognition of an epitope by an MHC II protein. We focus here on the human leukocyte antigen group DR (HLA-DR) of MHC II proteins, since they are the predominant isotype. HLA-DR proteins have a recognition groove whose pockets form energetically favorable interactions with specific side-chains of peptides approximately 9 residues in length. Numerous methods are available for epitope prediction, and they have been shown to be predictive of immunogenicity [25]. For the results, we employ two quite different and complementary methods.

#### ProPred

Sturniolo et al. [34] experimentally measured the binding affinity between individual residues and individual pockets of the MHC II binding groove on a limited set of alleles. They then created binding profiles for untested alleles through sequence and structure alignment with tested alleles. In this "pocket profile" method, TEPITOPE, the sum of position-specific weights for each residue in a 9-mer provides a score that is compared against a threshold to determine whether or not the peptide is in a given percentile of the best-recognized peptides. The approach was experimentally validated by comparing its predictions against HLA-DR selected and nonselected peptide repertoires; up to 80% of the selected peptides were correctly predicted at a threshold that yielded < 5% false positives. Singh and Raghava then built a tool, ProPred, to expand the scope of TEPITOPE and make it more easily accessible and applicable [29]. In a recent independent evaluation [25], ProPred did quite well in epitope prediction, achieving an average 0.73 area under the curve (AUC) across 14 different alleles. ProPred has also been successfully employed in a number of different studies; e.g., it has recently helped identify antigenic sites on a mosquito midgut glycoprotein, immunoreactive peptides in prostatic acid phosphatase, and promiscuous T-cell epitopes of three major secreted antigens of Mycobacterium tuberculosis [35-37]. In all three of these examples, ProPred facilitated the rapid identification of potential vaccine targets that were then experimentally characterized in detail. In our case study of Erythropoeitin (see Results), we found a quite striking match between ProPred predictions and published ELISPOT assay immunogenicity results.

#### SMM-align

Nielsen et al. [30] pursued a different approach to epitope prediction, developing the SMM-align method by applying machine learning techniques to large curated databases of experimentally validated epitopes: the Immune Epitope Database IEDB [38] and SYFPEITHI [39]. While ProPred uses data from single residues binding to single MHC II pockets, SMM-align uses data from whole peptides. Furthermore, while ProPred is based on sequence and structure alignment, SMM-align is uses Gibbs sampling and a regulated least squares regression to develop position specific scoring matrices that predict the binding affinity between an epitope and MHC II allele. In the independent evaluation mentioned above [25], SMM-align also achieved a mean 0.73 AUC (SMM-align and ProPred were the top two methods).

**Figure 2:**
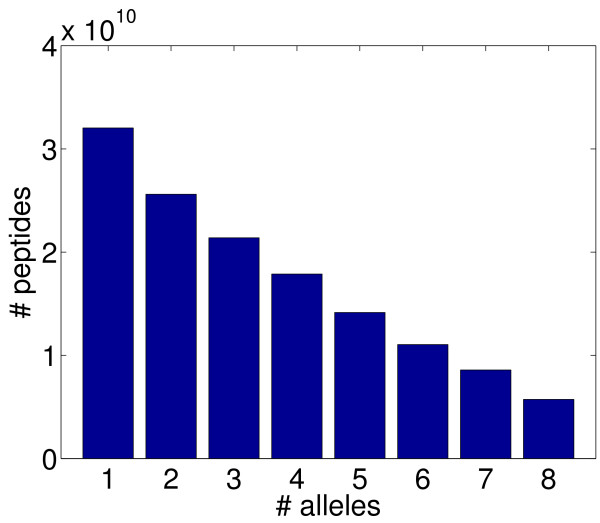
**Possible epitopes**. Number of 9-mer peptides (out of 20^9 ^possible) recognized by exactly the number of the eight common alleles we use for epitope scoring, relative to a 10% threshold (see text).

While there are over 50 different HLA-DR alleles, we have focused on 8 common alleles (DRB1*0101, DRB1*0301, DRB1*0401, DRB1*0701, DRB1*0801, DRB1*1101, DRB1*1301, and DRB1*1501) that represent the majority of human populations world-wide [40]. Thus our epitope score is the fraction of these 8 alleles predicted to recognize a peptide. In order to evaluate the potential for finding an epitope, we scored each of the 20^9 ^possible 9-mer peptides under ProPred at a 10% threshold. We found that 1.4 · 10^11 ^(26.63%) are predicted to be recognized by one or more alleles, including 5.7 · 10^9^(1.12%) by all 8 alleles; see Fig. 2 for a complete histogram.

### Stability evaluation

Evaluating the effects of mutations on a protein's stability and activity is at the heart of all rational protein engineering techniques. For the results, we consider three different methods using different sources of information to determine acceptable residues likely to maintain wild-type qualities.

#### BLOSUM

Given sequence alone, standard substitution tables such as BLOSUM [41] can evaluate the overall acceptability of a mutation, according to substitutions in sets of natural sequences. We compute a "relative" BLOSUM-62 score - the difference between the wild-type/wild-type score (diagonal) and the wild-type/mutant score. We obtain a reasonably conservative set of acceptable residues by only taking those with score differences of at most 4.

#### Conservation

A set of sequences related to the target protein reveals which positions are highly conserved, and to which amino acid(s), vs. which are more variable. In turn, this is indicative of which residues are riskier to mutate and which ones are safer. The utility of sequence alignments in engineering thermostablilized and functional protein variants has been proven in numerous experimental studies [42-46]. We use a multiple sequence alignment and phylogenetic tree to compute position-specific amino acid frequencies in a family. To avoid over-counting highly-related sequences, we weight sequences using a bottom-up tree-based algorithm [47]. The weighted position-specific score for amino acid *a *at position *i*, according to a multiple sequence alignment *F *of sequences *s *with (non-normalized) weights *w*_*s *_is then:(1)

We permit residues such that *ϕ*_*i*, *a *_exceeds a user-specified threshold, defaulting to -log 0.05 (i.e., 5% weighted frequency)

#### FoldX

When a structure is available, we employ the FoldX ΔΔG° predictor [48] to evaluate the change in free energy for each possible substitution. FoldX was demonstrated to achieve of 0.83 correlation between predicted and experimental ΔΔG° over 95% of a database after outlier removal. FoldX has since been successfully used to aid protein design, e.g., for custom DNA kinases and potential anticancer drugs [49,50]. It is important to note that our method does not need precise ΔΔG° prediction, but only an indication of whether a possible substitution is relatively "safe" (destabilizing by at most a little bit). We allow those residues whose predicted ΔΔG° values are at most a user-specified threshold, defaulting to 0.25 kcal/mol, more than the wild-type value (i.e., the mutant is nearly as good as, or even slightly better than, the wild-type).

Our problem specification treats substitutions independently of each other. While this is certainly a simplification, as residue interactions do affect stability and activity, it enables us to more quickly generate a number of solutions that are optimal (or near-optimal) with respect to epitope score. These solutions can then be subjected to more expensive analyses for non-additive effects.

### Dynamic programming algorithms

Given immunogenicity and stability predictions, represented in an epitope score *e *and set of allowed residues *M*, our goal (Problem 1) is to choose a set of mutations to minimize the total epitope score. In order to solve this problem by dynamic programming, let us define *T *[*i*, *X*] as the best possible total epitope score for the prefix of *S *ending at position *i*, such that the last 8 amino acids form the string *X*. *T *can be defined recursively:(2)

where · represents concatenation.

Optimal substructure holds: the best score ending at some position with some string must extend the best score ending at the previous position with a compatible string. Thus we can solve the recurrence by dynamic programming. Ultimately we want to find the minimum value in the last column (i.e., min_*X*_*T*[|*S*|, *X*]), and trace back to reconstruct the sequence. One small note of practical importance: when there is a tie for the minimum in Eq. 3, we should of course keep the wild-type amino acid.

The calculation for each cell requires constant time, and in the worst case there are *n *· 20^8 ^cells. However, in practice we only need to fill in the entries that use allowed substitutions; if these are reasonably conservative, the table is much smaller. In the BLOSUM-based approach described above, there are an average of 3.2 amino acids to consider for each position. The Results section provides position-by-position details for a specific protein, using BLOSUM, conservation, and FoldX.

In order to restrict the total number of substitutions made, an additional column can be added to the dynamic programming table. Now define *R*[*i*, *X*, *s*] as the best possible total epitope score for the prefix of *S *ending at position *i*, such that the last 8 amino acids form the string *X*, and that exactly *s *substitutions have been made from *S*. *R *can be defined recursively:(4)

where *I*{} is the indicator function, returning 1 if the predicate is true and 0 if it is false. Here we ensure that the *s *index of *R *counts the total number of substitutions, starting in the base case with the number in the N-terminal 8-mer, and then in the recursive case adding 1 iff the most C-terminal residue of *X *is different from the corresponding wild-type residue. The extension only affects the size of the table (scaled by a factor of *n*, unless *s *is restricted *a priori*); the cost for computing each cell remains constant. We can readily extend this approach to calculate an (integer) substitution score for each mutation, using *s *to track the total substitution score rather than the number of mutations.

While a standard dynamic programming backtrace returns a single optimal solution, there may in fact be multiple variants with the same score. It may also be beneficial to consider near-optimal variants, as it is unlikely that our epitope score and evaluation of mutations are perfect, and thus near-optimal variants are worth considering. Upon finding the set of optimal and near-optimal solutions, we can subject them to further analysis, e.g., to model the effects of multiple substitutions, or to consider the ease of construction. Furthermore, by comparing and contrasting the good variants, we can better assess the robustness of a variant (do similar substitution patterns show up among the good ones?), as well as the general utility of a substitution (does it show up in many good variants?).

The problem of extracting multiple optimal and near-optimal solutions in dynamic programming has been extensively studied, from the early days of the field [51]. It has also received attention specifically in the bioinformatics community, as dynamic programming is at the heart of sequence alignment (among other significant problems). For example, Waterman and Byers [52] modified the standard dynamic programming backtracing procedure to produce near-optimal solutions, Naor and Brutlag [53] presented an alternative approach for representing (rather than enumerating) all alignments whose score is within a factor of optimal, and Gusfield [54] explicitly accounted for the objective function parameters that yield different optimal solutions.

Our current implementation employs the approach described by Waterman and Byers [52] in order to generate multiple possible variants.

### Implementation

We have implemented our method in platform-independent Java code. The program takes as input a target protein sequence, along with specifications of how to evaluate stability and immunogenicity. As discussed above, the program can evaluate stability with BLOSUM, conservation (given the family multiple sequence alignment and phylogenetic tree), or FoldX (given the position-specific ΔΔG° values output from that program), and immunogenicity with ProPred (at a user-specified 1-10% threshold) or SMM-align (at a user-specified IC_50 _from 50-5000). The user must indicate which methods to employ, along with any necessary inputs (MSA and tree, or FoldX output) and can adjust the thresholds for acceptable stability scores (defaults are provided as described above). The program outputs all tied-for-optimal and near-optimal variants up to a user-specified limit, along with stability and immunogenicity evaluations of each variant according to the various predictors.

The software can be freely obtained for academic use by request from the authors. A demonstration web-based version is available at http://www.cs.dartmouth.edu/~cbk/deimm/.

## Results and Discussion

We demonstrate our approach by applying it to a number of proteins that have been the object of previous deimmunization efforts. We explore the favorable sequence space of these proteins by evaluating epitope score under the ProPred method at a 10% threshold, and considering allowed residues under one of BLOSUM, conservation, or FoldX. We then independently assess each variant under SMM-align for epitope score and each of the other measures for stability.

In presenting stability predictions, we separately sum the value of each metric (BLOSUM, conservation, FoldX) over all the chosen substitutions. This enables assessment of a plan under different and potentially complementary measures; developing a consensus method in the future might yield even better results. The BLOSUM score for each substitution is either 0 (allowed) or 1 (disallowed). The negative-log conservation score for a substitution ranges from roughly 0.01 to 4.61 (99% to 1% weighted frequency), with a maximum of roughly 3 (5% weighted frequency) for allowed substitutions. For FoldX, the score for a substitution ranges from roughly -3 to 3 (negative implies stabilizing), with a maximum of 0.25 for allowed substitutions.

### Staphylokinase (SakSTAR)

Warmerdam et al. [19] sought to deimmunize the fibrin-selective thrombolytic agent staphylokinase, specifically the SakSTAR wild-type variant derived from a lysogenic *S. aureus *strain. They targeted the C3 region, spanning residues 71-87, which was recognized by 90% of the T-cells cloned from a set of donors. Based on results from alanine scanning mutagenesis, sets of 2-4 alanine substitutions were selected to produce new variants designed to reduce immunogenicity.

**Figure 3:**
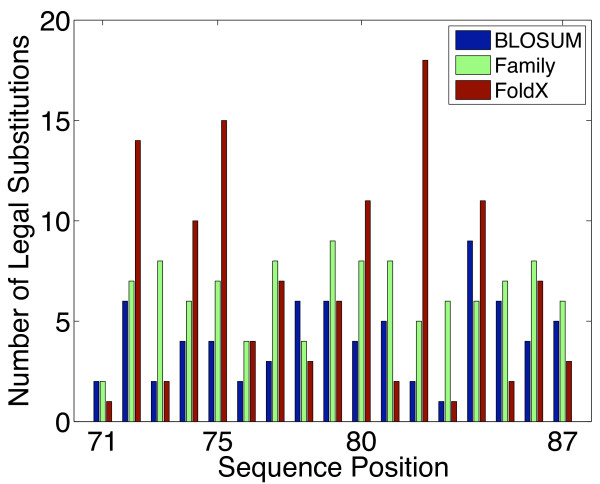
**Position-specific allowed residues in SakSTAR peptide**. Number of allowed residues for each position of SakSTAR 71-87 by BLOSUM, conservation, and FoldX.

We applied our approach to the original wild-type 71-87 peptide, using the Staphylokinase/Streptokinase family (Pfam accession PF02821) for conservation statistics and SakSTAR crystal structure (pdb id 2SAK) for FoldX calculations. Fig. 3 shows the amount of freedom in planning, in terms of the number of allowable residues at each position under our three evaluation methods. BLOSUM is typically more conservative and is overall more uniform; conservation depends on the position-specific diversity in the family; and FoldX allows more mutations when analysis of the structure at hand indicates that they would not be too destabilizing. On average, BLOSUM permits 4.2 residues per position, conservation 6.4, and FoldX 6.9. Table 1 summarizes some of our optimized variants, one per allowed residue predicate (BLOSUM, conservation, and FoldX). Our objective function is the number of ProPred-predicted epitopes, so this number naturally decreases with the number of substitutions, though it is worth noting that each substitution actually deletes several predicted epitopes. Furthermore, the independent predictor SMM-align (not part of the objective function) likewise trends downward with an increasing number of substitutions. Since ProPred was derived from pocket profiles and sequence alignments, while SMM-align was trained on specific experimentally identified epitopes, they provide complementary assessments of immunogenicity, and their agreement suggests that we are indeed likely to be deleting actual epitopes. By comparing results for the different allowed residue predicates, we can gain insights into how best to delete these epitopes, from a stability-preservation viewpoint. For example, we see that V79 was chosen for the first substitution under all three approaches. With BLOSUM, the conservative V79T was chosen; with conservation, D79 was recognized as sufficiently common in the sequence record; and with FoldX, K79 was predicted to maintain stability. On the other hand, the three-substitution conservation-based variant eliminates all epitopes (and of course looks good from a conservation analysis), but incurs a large ΔΔG° penalty relative to the solutions from the other metrics. It is worth noting that currently only the epitope score is the objective function (though we could readily employ a linear combination with a substitution score), and the goal is to delete as many epitopes as possible using substitutions allowed by a particular predicate. Thus, for example, in order to delete more epitopes, a conservation-based design may actually end up with a larger conservation penalty than a BLOSUM-based design, by using less common substitutions (but ones still meeting the weighted 0.05% frequency threshold) that are not allowed by BLOSUM. Further insights can be gained by considering all tied-for-optimal variants (Additional file 1, Table S1). For example, we can identify commonly selected mutations, e.g., V79T and V79K, and might consider variants incorporating them to be of higher quality.

**Table 1:** SakSTAR 71-87 Peptide.

Variant	SakSTAR 71-87	*E*	*B*	*C*	ΔΔG°	*S*
wild type	TAYKEFRVVELDPSAKI	16	0	0.00	0.00	9
**1 substitution**						
Warmerdam	......A..........	12	1	0.75	0.43	7
BLOSUM	........T........	8	0	-0.74	1.13	5
Conservation	........D........	5	1	0.72	1.90	3
FoldX	........K........	9	1	0.94	0.00	5
**2 substitutions**						
Warmerdam	......A..A.......	15	2	2.48	1.00	11
BLOSUM	........T.F......	5	0	0.28	3.12	2
Conservation	.....K..D........	1	2	0.51	4.14	1
FoldX	........K....T...	5	1	2.43	0.18	6
**3 substitutions**						
Warmerdam	......A..A.A.....	20	3	4.96	0.22	14
BLOSUM	...E....T.F......	3	0	0.45	3.73	1
Conservation	...D.K..D........	0	3	1.46	4.52	0
FoldX	...A....K....T...	3	2	3.44	0.08	4
**4 substitutions**						
Warmerdam	...Q..S..S....S..	15	2	4.37	2.12	7
BLOSUM	......QMA.F......	1	0	3.91	4.66	2
Conservation	n/a					
FoldX	...AD...K....T...	2	2	1.50	-0.30	4

Variants of SakSTAR 71-87 by Warmerdam et al. B1919, as well as by our dynamic programming algorithm, optimizing for ProPred and allowing mutations according to BLOSUM, conservation, or FoldX. *E*: number of ProPred epitopes; *B*, *C*, and ΔΔG°: total substitution penalty under BLOSUM, conservation, and FoldX, resp.; *S*: number of SMM-align epitopes.

**Figure 4:**
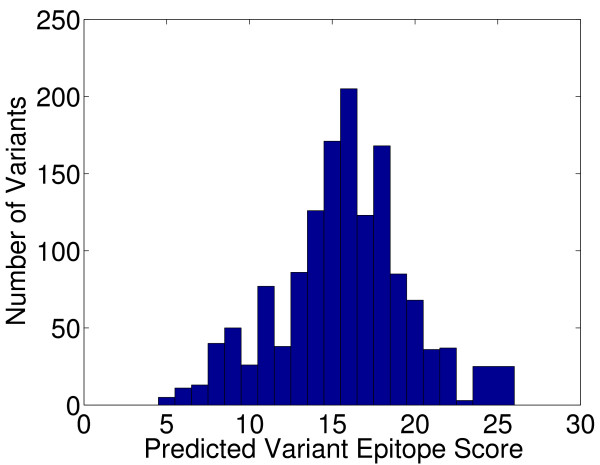
**Exhaustive 2-mutation search scores for SakSTAR peptide**. Histogram of predicted epitope scores for all 2-mutation variants of SakSTAR 71-87 under BLOSUM.

Our method identifies the favorable region of the sequence space, but a natural question is what portion of the space is favorable. In other words, are many or most variants likely to be good anyway? Fig. 4 shows the distribution of epitope scores for all 2-mutation variants of SakSTAR, using all acceptable mutations according to the BLOSUM evaluation. (Of course, with larger numbers of mutations and longer sequence, the exhaustive approach would not be feasible.) The figure makes clear that most variants have scores much worse than the optimal ones designed by our approach: the median score is 16 and only 5 of the 1338 sequences (0.37%) achieve the optimal score of 5. Thus experiment planning techniques are required, as stochastic methods are unlikely to produce high-quality variants.

Our designs show dramatic reduction in predicted T-cell epitope content (under both ProPred and SMM-align) compared to the variants chosen by Warmerdam et al. Their variants minimally decrease, or even introduce new predicted T-cell epitopes, due in part to limitations in their selection of amino acids (using only alanine for the 2- and 3-substitution variants).

**Figure 5:**
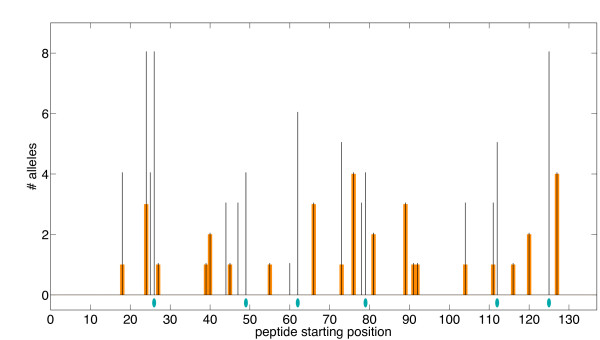
**Full-length SakSTAR variant profile**. Optimized 6-substitution full-length SakSTAR variant with ProPred epitope scoring and conservation-based substitutions. *x*-axis: starting position of each 9-mer; *y*-axis: predicted number of alleles recognizing the 9-mer. Thin black bars indicate wild-type scores and thick orange bars indicate variant scores. Note: wild-type epitope scores are always greater than or equal to corresponding variant ones; i.e., we never introduce new epitopes. Blue ellipses indicate mutated positions (refer to Table 2).

While Warmerdam et al. focused effort on the C3 region, our method is able to globally optimize an entire protein and thereby address a weakness identified in the earlier method: the "vast majority of humans recognize additional immunogenic SakSTAR regions" [19]. Fig. 5 profiles a 6-mutation full-protein variant identified by our method. Notice that even though it was not specifically targeted, the C3 immunogenic region was addressed with substitution V79D. In addition, mutations were selected in five other regions of high predicted immunogenicity. Each mutation deletes an average of 6.5 epitopes, overlapping the substituted position and/or for different MHC-II alleles. Furthermore (Table 2), all substitutions are to amino acids with weighted frequency greater than .05 at those positions in the staphylokinase family. Table 2 and Additional file 1, Table S2 detail a number of the full-protein variants for different numbers of mutations. Again the SMM-align epitope evaluation correlates very well with the optimized ProPred score, trending downward with increasing numbers of substitutions. The different allowed residue predicates all hit the C3 region (71-87) within the first few substitutions (again often picking V79), but also delete epitopes in a number of other predicted immunogenic regions (see again Fig. 5). The designs compare favorably with the Warmerdam designs in terms of both epitope predictors. The conservation-based variants tend to be particularly aggressive in deleting epitopes by choosing other residues represented in the family, but sacrifice more in predicted stability under FoldX.

**Table 2:** Full-length SakSTAR.

Variant	SakSTAR 1-136	*E*	*B*	*C*	ΔΔG°	*S*
wild type	SakSTAR	99	0	0.00	0.00	49
**1 substitution**						
Warmerdam	R77A	95	1	0.75	0.43	47
BLOSUM	V27T	88	0	0.89	2.08	48
Conservation	M26D	79	1	1.15	1.89	41
FoldX	V112P	90	1	0.93	-0.03	47
**2 substitutions**						
Warmerdam	R77A,E80A	98	2	2.48	1.00	51
BLOSUM	V27T,S84E	79	0	3.18	2.70	43
Conservation	M26D,V79D	68	2	1.87	3.79	35
FoldX	Y24H,V112P	82	2	1.89	0.04	43
**3 substitutions**						
Warmerdam	R77A,E80A,D82A	105	3	4.96	0.22	55
BLOSUM	V27T,S84E,V112A	70	0	1.98	3.16	41
Conservation	M26D,I49D,V79D	58	3	1.51	7.46	31
FoldX	Y24H,V79K,V112P	75	3	2.83	0.04	39
**4 substitutions**						
Warmerdam	K74Q,R77S,E80S,D82S	103	3	5.93	1.28	52
BLOSUM	V27T,V45A,S84E,V112A	63	0	2.36	3.98	42
Conservation	M26D,I49D,V79D,V112K	49	4	1.28	6.72	29
FoldX	Y24H,N28E,V79K,V112P	69	4	4.28	0.00	35
**5 substitutions**						
BLOSUM	V27T,V45A,S84E,V112A,K130E	58	0	3.12	5.41	40
Conservation	M26D,I49D,V79D,V112K,F125E	41	5	1.74	12.60	25
FoldX	Y24H,N28E,Y62R,V79K,V112P	63	5	4.37	0.12	33
**6 substitutions**						
BLOSUM	V27T,V45A,V79T,V89T,V112A,K130E	54	0	0.74	8.26	41
Conservation	M26D,I49D,Y62D,V79D,V112K,F125E	34	6	1.92	13.56	23
FoldX	Y24H,N28E,Y62K,V79K,S84T,V112P	58	5	5.32	0.01	34

Variants of the full SakSTAR protein by Warmerdam et al. [19], as well as by our dynamic programming algorithm, optimizing for ProPred and allowing mutations according to BLOSUM, conservation, or FoldX. *E*: number of ProPred epitopes; *B*, *C*, and ΔΔG°: total substitution penalty under BLOSUM, conservation, and FoldX, resp.; *S*: number of SMM-align epitopes.

#### ProPred Threshold

**Figure 6:**
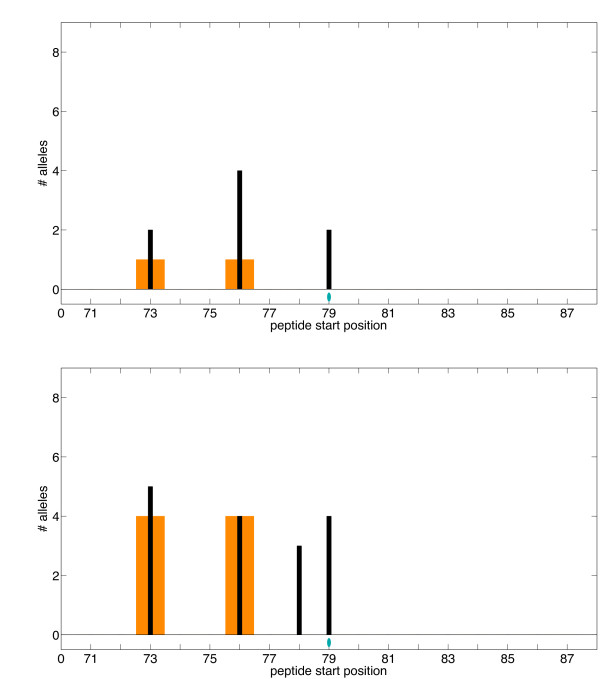
**SakSTAR peptide with ProPred 5% and 10% thresholds**. Optimized SakSTAR peptide variant with ProPred epitope scoring at 5% (top) and 10% (bottom) thresholds. *x*-axis: starting position of each 9-mer; *y*-axis: predicted number of alleles recognizing the 9-mer. Thin black bars indicate wild-type scores and thick orange bars indicate variant scores. Note: wild-type epitope scores are always greater than or equal to corresponding variant ones; i.e., we never introduce new epitopes. The blue ellipse indicates BLOSUM-based substitution V79T.

Epitope predictors employ thresholds in deciding to label peptides as MHC-II binders or non-binders. To illustrate our algorithm, we have employed the "loosest" ProPred threshold of 10%, erring on the side of predicting spurious epitopes instead of on the side of missing epitopes. We also evaluated plans for SakSTAR based on a tighter threshold of 5%. As expected, with the 5% threshold, ProPred predicts fewer epitopes than with the 10% threshold: SakSTAR 71-87 has 16 predicted epitopes at 10% but only 8 at 5%. At 5% our algorithm finds completely deimmunized variants for the peptide within 4 substitutions (Additional file 1, Table S3). The substitution V79T eliminates 75% of the epitopes predicted in the 71-87 peptide at the 5% threshold and 50% of those predicted at the 10% threshold (Fig. 6). For full-length SakSTAR, both thresholds predict the same regions to be immunodominant (Fig. 5 and Additional file 1, Fig. S1). Changing the threshold from 10% to 5% seems to evenly attenuate the epitope signal across the protein. Of particular significance, we note that our optimization algorithm selects exactly the same full-length 6-substitution conservation-based variant with the 5% threshold (Additional file 1, Table S4) as it did for 10% (Table 2). The plan eliminates a strikingly high proportion of epitopes, 66% at the 10% threshold and 88% at 5%.

#### Allele Analysis

**Figure 7:**
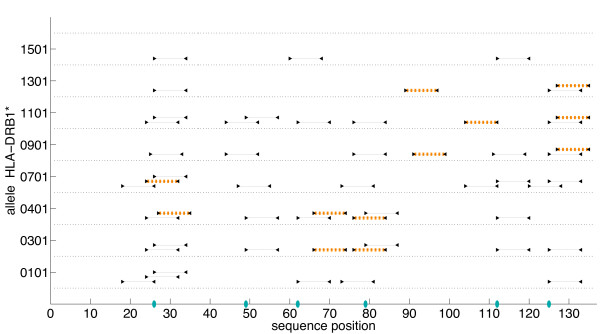
**Full-length SakSTAR epitope analysis by allele with ProPred 5% threshold**. Optimized 6-substitution SakSTAR variant with ProPred epitope scoring (5% threshold) and conservation-based substitutions. *x*-axis: sequence position; *y*-axis: HLA-DRB1* allele recognizing the 9-mer. Lines: 9-residue extent of epitopes in the wild-type; cross-hatched lines: epitopes remaining in the variant. Blue ellipses indicate mutated positions (refer to Additional File 1 Table S4).

**Figure 8:**
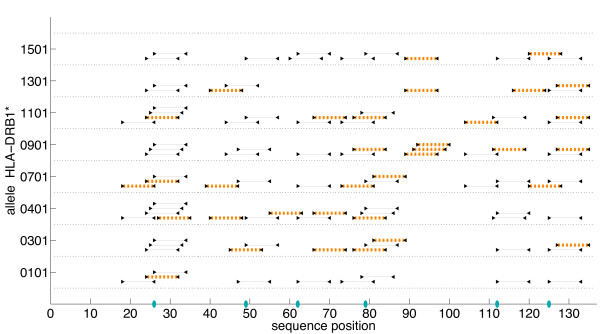
**Full-length SakSTAR epitope analysis by allele with ProPred 10% threshold**. Optimized 6-substitution SakSTAR variant with ProPred epitope scoring (10% threshold) and conservation-based substitutions. *x*-axis: sequence position; *y*-axis: HLA-DRB1* allele recognizing the 9-mer. Lines: 9-residue extent of epitopes in the wild-type; cross-hatched lines: epitopes remaining in the variant. Blue ellipses indicate mutated positions (refer to Table 2).

A detailed analysis of predicted SakSTAR epitopes by binding allele shows that our 6-substitution conservation-based variant eliminates some of the epitopes predicted for each different allele (Figs. 7 and 8). At the ProPred 5% threshold, our design eliminates all epitopes predicted to bind to alleles HLA-DRB1*0101 and HLA-DRB1*1501. Total allele elimination does not occur at the 10% threshold, although in the variant, alleles 0101 and 1501 are predicted to bind only 1 and 2 epitopes, respectively. The plots further underscore the observation that the 5% and 10% thresholds yield similar epitope profiles across the whole protein both by sequence and by allele. As mentioned above, the optimal deimmunizing mutations are identical for plans under both thresholds, but a greater percentage of predicted epitopes are eliminated at the 5% threshold. In general, it is easier to eliminate an epitope that lies between the 5% and 10% threshold than one that exceeds the 10% threshold. For example, in the 5% plan, the V79T mutation eliminates 3 of 4 epitopes beginning at residue 76, but none of these four epitopes are eliminated at the 10% threshold.

### Erythropoietin (Epo)

Tangri et al. [22] focused on two regions in the protein therapeutic erythropoietin (Epo), residues 101-115 and 136-150, which they experimentally determined to be immunogenic during an intensive analysis of peptide fragments spanning the entire length of the protein. They engineered four variants targeting the anchor residues of identified T-cell epitopes in these regions: L102P/S164D (named G2), T107D/S146D (G3), L102G/T107D/S146D (G4), and L102S/T107D/S146D (G5).

**Figure 9:**
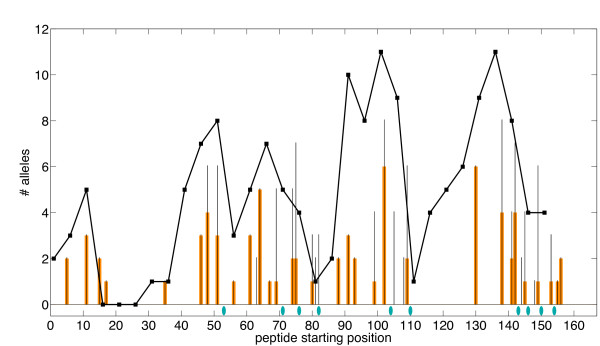
**Full-length Epo variant profile**. Optimized 10-substitution Epo variant with ProPred epitope scoring and BLOSUM-based substitutions. *x*-axis: starting position of each 9-mer; *y*-axis: predicted number of alleles recognizing the 9-mer. Thin black bars indicate wild-type scores and thick orange bars indicate variant scores. Note: wild-type epitope scores are always greater than or equal to corresponding variant ones; i.e., we never introduce new epitopes. Blue ellipses indicate mutated positions (refer to Table 3). The line plot, from Tangri et al. [22] displays wild-type Epo antigenicity using ELISPOT assays, with black squares giving the number of alleles bound to overlapping 15-mers.

We applied our methods to explore the favorable sequence space of Epo, using the Erythropoietin/thrombopoietin family (Pfam accession PF00758) for the conservation statistics and the crystal structure of human Epo (pdb id 1EER) for the FoldX analysis. As demonstrated above for SakSTAR, our method is not restricted to optimizing only targeted regions, but can instead seek to delete epitopes throughout the protein. Since both the ProPred and SMM-align epitope predictors and Tangri et al.'s *in vitro *assays showed that there are many immunogenic regions in Epo, we performed full-protein optimization, rather than restricting the allowed substitutions to the 101-115 and 136-150 regions. Fig. 9 illustrates a 10-mutation BLOSUM-based variant. The black line is an experimentally determined immunogenicity plot from Tangri et al. [22] and trends well with the ProPred model of immunogenicity. Some deviations may be explained by the difference in alleles tested (we share 6 of their alleles), and by the fact that they analyze 15-mers at every 5 positions while we analyze 9-mers at every position. Nonetheless, the correlation is quite striking, as is the ability of our design to target most of the highly immunogenic regions with only a small number of substitutions. Each substitution is quite effective, deleting an average of 6.3 epitopes.

Table 3 summarizes a number of our optimized variants, as with SakSTAR listing just one for each allowed residue predicate (see Additional file 1, Table S5 for a full list). The first substitution made under BLOSUM and conservation is to R110, in the 101-115 region, while that under FoldX is to N147, in the 136-150 region, though neither of these regions was specifically targeted. As more substitutions are added, other predicted epitopes are deleted, including more in those regions. Thus our objective function, the ProPred score, continues to decrease; the trend is roughly the same for both the Tangri variants and ours. In some cases the independent SMM-align score fluctuates more than others, e.g., BLOSUM alternates between using S164D or not. This observation highlights the fact that some substitutions may be particularly good for the SMM-align score but not as important for the ProPred objective function.

**Table 3:** Full-length Epo.

Variant	Epo 1-166	*E*	*B*	*C*	ΔΔG°	*S*
wild type	Epo	136	0	0.00	0.00	74
**1 substitution**						
BLOSUM	R110Q	126	0	2.87	0.70	72
Conservation	R110G	128	1	2.81	1.87	75
FoldX	N147D	124	1	2.22	-0.33	70
**2 substitutions**						
Tangri G2	L102P,S146D	118	1	4.97	6.06	65
Tangri G3	T107D,S146D	121	1	4.67	1.02	62
BLOSUM	R110Q,S146D	119	0	4.92	0.92	65
Conservation	R76A,R110G	120	2	5.48	3.47	76
FoldX	F48D,N147D	115	2	5.07	-0.59	65
**3 substitutions**						
Tangri G4	L102G,T107D,S146D	113	2	7.59	6.09	57
Tangri G5	L102S,T107D,S146D	115	2	7.59	4.89	58
BLOSUM	R76Q,V82T,R110Q	111	0	8.91	1.78	72
Conservation	V56E,R76A,R110G	113	3	7.95	3.29	69
FoldX	F48D,V82K,N147D	108	3	6.96	-1.18	63
**4 substitutions**						
BLOSUM	R76Q,V82T,R110Q,S146D	104	0	10.96	2.00	65
Conservation	V56E,R76A,V82A,R110G	106	3	7.62	3.06	68
FoldX	F48D,V82E,L93E,N147D	101	4	10.01	-0.87	60
**5 substitutions**						
BLOSUM	S71E,R76K,V82T,R110Q,S146D	98	0	12.91	1.31	62
Conservation	V56E,R76A,V82A,L91G,R110G	101	4	10.40	4.77	66
FoldX	F48D,V82E,L93E,S146D,N147D	95	4	12.05	-0.65	57
**10 substitutions**						
BLOSUM	R53Q,S71D,R76K,V82T,S104D,	73	0	23.90	2.93	49
	R110Q,R143Q,S146D,R150Q,K154E					

Variants of the entire Epo protein by Tangri et al. [22] as well as by our dynamic programming algorithm, optimizing for ProPred and allowing mutations according to BLOSUM, conservation, or FoldX. *E*: number of ProPred epitopes; *B*, *C*, and ΔΔG°: total substitution penalty under BLOSUM, conservation, and FoldX, resp.; *S*: number of SMM-align epitopes.

As with SakSTAR, comparison of the different predicates yields insights into positions and substitutions that appear to be good in general; e.g., V82T under BLOSUM, V82A under conservation, and V82E under FoldX, deleting 7, 6, and 7 epitopes respectively. Notably, none of the V82 mutations eliminates the epitope anchored at L80 on allele HLA-DRB*0401. Otherwise V82T and V82A eliminate all of the epitopes in the region overlapping position 82. Our global optimization recognizes diminishing returns at this area on the protein. While adding additional mutations in the region may eliminate the final regional epitope at L80; it is only one epitope, and mutations elsewhere eliminate more epitopes.

### Therapeutic Antibodies

Lazar et al. [23] introduced the concept of "human string content," or the percent identity between peptides derived from a test antibody sequence and corresponding peptides taken from a multiple sequence alignment of homologous human antibodies. We applied our methodology to anti-Her2/neu antibody 4D5, the anti-EGFR antibody 225, and the anti-EpCAM antibody 17-1A. At the 16-substitution level, we are able to reduce epitope score by about 70-90%; this compares favorably to the previous work, which required more than four times that many substitutions. See Additional file 1 for a more detailed description of the case study and our results.

## Conclusions

We have shown that dynamic programming can address the problem of designing protein variants predicted to have reduced immunogenicity while maintaining stability. Our method found a number of variants that compare favorably to those developed in previous efforts. In many cases, our designs delete more epitopes than previous efforts, as measured by the ProPred pocket profile method and independently assessed with the SMM-align method. At the same time, the capacity of our algorithm to integrate stability analysis with deimmunization resulted in variants predicted to maintain greater thermodynamic stability. We further showed our optimization methods to be highly efficient, eliminating on average over 6 epitopes per mutation. Finally, one of the most powerful features of our methods is that we achieve global deimmunization as opposed to targeted deletion of a single epitope regardless of other immunogenic or functional consequences.

The algorithm guarantees that our variants are provably optimal with respect to the epitope and stability predictors, but this does not guarantee optimal properties *in vivo*. Instead, our algorithm should be viewed as a way to suggest variants worth studying experimentally. It provides a tool for the protein designer to explore the space of designs and focus in what appears to be a beneficial region, according to the best available predictions.

Future experimental work will focus on selection of one or more therapeutic targets that will be subjected to an exhaustive optimization under several mutational loads. Based on the resulting plans, small libraries of candidate variants will be constructed, expressed and purified, tested for functionality, and experimentally evaluated for immunogenic potential. Further computational work will develop other classes of optimization algorithms for incorporating properties not strictly local in terms of the primary sequence, such as residues that covary in the sequence record or form strong interactions in the three-dimensional structure.

## Availability and requirements

**Project name: **DP^2^: dynamic programming for deimmunizing proteins

**Project home page: **http://www.cs.dartmouth.edu/~cbk/deimm/

**Operating system(s): **Platform independent

**Programming language: **Java

**Other requirements: **Java 1.6 or higher

**License: **GNU GPL

**Any restrictions to use by non-academics: **Please contact the authors before non-academic use.

## Authors' contributions

ASP, KEG, and CBK developed the approach; ASP, WZ, and CBK designed the algorithms, ASP implemented the algorithms and collected the results; ASP, KEG, and CBK analyzed the results and wrote the paper. All authors read and approved the final manuscript.

## Supplementary Material

Additional file 1 **Additional variants**. The file includes additional variants for SakSTAR 71-87, full-length SakSTAR, and full-length Epo, as well as an additional case study for Abs 4D5, 225, and 17-1A. Click here for file
