# Supplementary material for: Optimization algorithms for functional deimmunization of therapeutic proteins
Source: BMC Bioinformatics. 2010 Apr 9;11:180. doi: 10.1186/1471-2105-11-180 (PMC2873530; doi:10.1186/1471-2105-11-180)
Supplement: Additional file 1 — Additional variants. The file includes additional variants for SakSTAR 71-87, full-length SakSTAR, and full-length Epo, as well as an additional case study for Abs 4D5, 225, and 17-1A. [file 1471-2105-11-180-S1.PDF]

Optimization algorithms for functional deimmunization of therapeutic proteins
Parker, Zheng, Griswold, Bailey-Kellogg
Additional file 1

This supplementary file includes additional variants for SakSTAR 71–87, full-length SakSTAR, and full-length Epo, as well as an additional case study for Abs 4D5, 225, and 17-1A.

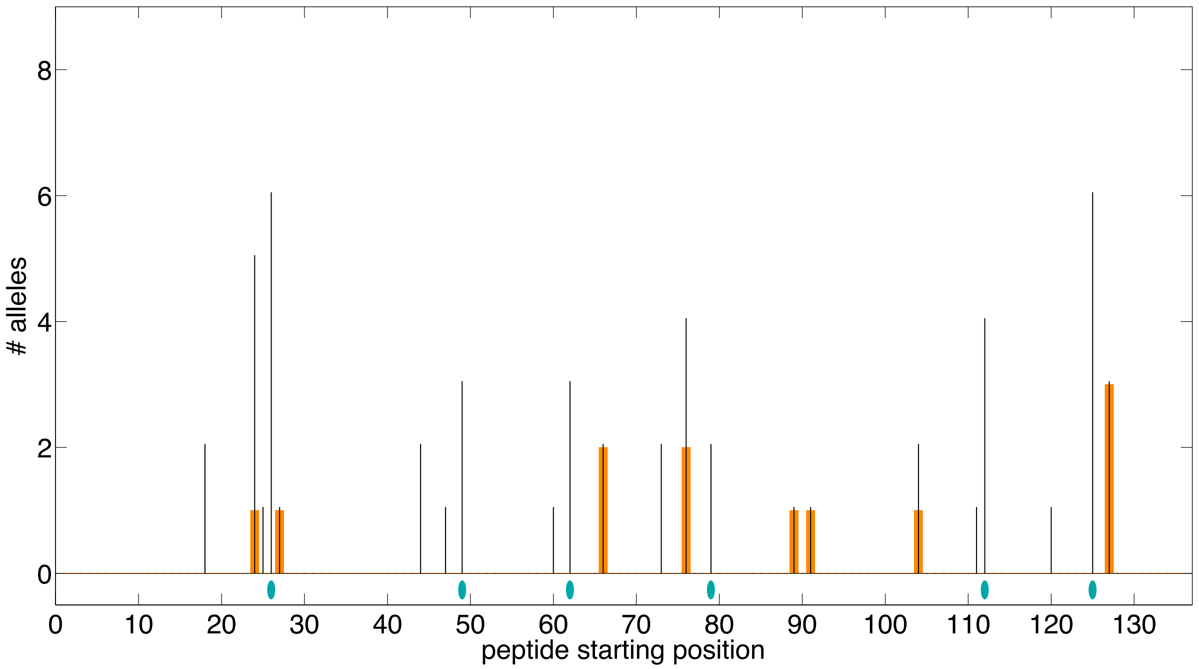

Figure S1: Optimized 6-substitution full-length SakSTAR variant with ProPred epitope scoring (5% threshold) and conservation-based substitutions. x-axis: starting position of each 9-mer; y-axis: predicted number of alleles recognizing the 9-mer. Thin black bars indicate wild-type scores and thick orange bars indicate variant scores. Note: The same regions are immunodominant as with the 10% threshold and the algorithm computes the same optimal plan (main text, Fig. 5). Blue ellipses indicate mutated positions (refer to Table S4).

Table S1: All tied-for-optimal variants of SakSTAR 71–87, using BLOSUM, conservation, or FoldX to select acceptable mutations. E: number of epitopes; B, C, and $\Delta\Delta G°$: total substitution penalty under BLOSUM, conservation, and FoldX, resp; S: number of SMM-align epitopes.

Variant	SakStar 71–87	E	B	C	$\Delta\Delta$G°	S
wild type	TAYKEFRVVELDPSAKI	16	0	0.00	0.00	9
wild type	TAYKEFRVVELDPSAKI	16	0	0.00	0.00	9
	**1 substitution**					
BLOSUM	........T........	8	0	-0.74	1.13	5
Conservation	........D........	5	1	0.72	1.90	3
FoldX	........K........	9	1	0.94	0.00	5
	**2 substitutions**					
BLOSUM	.......A.....E...	5	0	3.31	3.32	3
BLOSUM	........T....G...	5	0	1.54	1.82	3
BLOSUM	........T.F......	5	0	0.28	3.12	2
BLOSUM	.......AT........	5	0	0.28	3.83	5
BLOSUM	......K.T........	5	0	-0.66	0.94	4
Conservation	.....K..D........	1	2	0.51	4.14	1
FoldX	........K....T...	5	1	2.43	0.18	6
	**3 substitutions**					
BLOSUM	...E....T....G...	3	0	1.71	2.43	2
BLOSUM	.......AT.F......	3	0	1.31	5.82	2
BLOSUM	...E....T.F......	3	0	0.45	3.73	1
BLOSUM	...E...TT........	3	0	0.45	3.62	5
BLOSUM	...E..K.T........	3	0	-0.49	1.55	3
Conservation	...D.K..D........	0	3	1.46	4.52	0
Conservation	..H..K..D........	0	3	-0.48	6.60	0
Conservation	...P.K..D........	0	3	-0.83	5.01	0
Conservation	.....K.YD........	0	3	1.33	6.77	1
Conservation	...D.K..E........	0	3	1.46	3.75	0
Conservation	...P.K..E........	0	3	-0.83	4.24	0
Conservation	...D.K..Q........	0	3	1.48	3.25	1
FoldX	...A....K....T...	3	2	3.44	0.08	4
	**4 substitutions**					
BLOSUM	......KMA.F......	1	0	3.07	3.87	4
BLOSUM	......QMA.F......	1	0	3.91	4.66	2
Conservation	n/a					
FoldX	...AD...K....T...	2	2	1.50	-0.30	4
FoldX	...A.W..K....T...	2	3	4.47	-0.62	5

Table S2: Variants of the full-length SakSTAR protein by Warmerdam et al., as well as by our dynamic programming algorithm, optimizing for ProPred and allowing mutations according to BLOSUM, conservation, or FoldX. E: number of ProPred epitopes; B, C, and $\Delta\Delta G°$: total substitution penalty under BLOSUM, conservation, and FoldX, resp.; S: number of SMM-align epitopes.

Variant	SakSTAR 1–136	E	B	C	$\Delta\Delta G°$	S
wild type	`SakSTAR`	99	0	0.00	0.00	49
	**1 substitution**					
Warmerdam	`R77A`	95	1	0.75	0.43	47
BLOSUM	`V27T`	88	0	0.89	2.08	48
Conservation	`M26D`	79	1	1.15	1.89	41
FoldX	`V112K`	90	1	-0.23	-0.74	47
	**2 substitutions**					
Warmerdam	`R77A,E80A`	98	2	2.48	1.00	51
BLOSUM	`V27T,V112A`	79	0	-0.30	2.54	46
BLOSUM	`V27T,S84E`	79	0	3.18	2.70	43
Conservation	`M26D,V79D`	68	2	1.87	3.79	35
FoldX	`Y24H,V112D`	82	2	1.89	-0.53	43
FoldX	`Y24H,V112K`	82	2	0.73	-0.67	43
	**3 substitutions**					
Warmerdam	`R77A,E80A,D82A`	105	3	4.96	0.22	55
BLOSUM	`V27T,S84E,V112A`	70	0	1.98	3.16	41
Conservation	`M26D,I49D,V79D`	58	3	1.51	7.46	31
FoldX	`Y24H,V79K,V112D`	75	3	2.83	-0.53	39
FoldX	`Y24H,V79K,V112K`	75	3	1.67	-0.67	39
	**4 substitutions**					
Warmerdam	`K74Q,R77S,E80S,D82S`	103	3	5.93	1.28	52
BLOSUM	`V27T,V45A,S84E,V112A`	63	0	2.36	3.98	42
Conservation	`M26D,I49D,V79D,V112A`	49	3	0.31	7.92	29
Conservation	`M26D,I49D,V79D,V112K`	49	4	1.28	6.72	29
FoldX	`Y24H,Y62K,V79K,V112D`	69	4	2.38	-0.70	37
FoldX	`Y24H,N28E,V79K,V112D`	69	4	4.28	-0.57	35
	**5 substitutions**					
BLOSUM	`V27T,V45A,S84E,V112A,K130E`	58	0	3.12	5.41	40
Conservation	`M26D,I49D,V79D,V112A,F125E`	41	4	0.77	13.80	25
Conservation	`M26D,I49D,V79D,V112K,F125E`	41	5	1.74	12.60	25
FoldX	`Y24H,N28E,Y62K,V79K,V112D`	63	5	3.83	-0.74	33
FoldX	`Y24H,N28E,Y62Q,V79K,V112E`	63	5	4.47	-0.46	33
FoldX	`Y24H,N28E,Y62K,V79K,V112K`	63	5	2.67	-0.88	33
	**6 substitutions**					
BLOSUM	`V27T,V45A,S84E,K94E,V112A,K130E`	54	0	3.13	5.74	40
BLOSUM	`V27T,V45A,V79T,V89T,V112A,K130E`	54	0	0.74	8.26	41
BLOSUM	`V27T,V45A,V78A,S84E,V112A,K130E`	54	0	4.15	8.11	39
Conservation	`M26D,I49D,Y62D,V79D,V112A,F125E`	34	5	0.95	14.76	23
Conservation	`M26D,I49D,Y62D,V79D,V112E,F125E`	34	6	2.87	13.93	23
Conservation	`M26D,I49D,Y62D,V79D,V112K,F125E`	34	6	1.92	13.56	23
FoldX	`Y24H,N28E,G31D,Y62E,V79K,V112D`	58	6	5.50	-0.48	32
FoldX	`Y24H,N28E,G31D,Y62R,V79K,V112E`	58	6	5.00	-0.26	32
FoldX	`Y24H,N28E,Y62K,V79K,S84T,V112K`	58	5	4.16	-0.70	34
FoldX	`Y24H,N28E,G31D,Y62R,V79K,V112P`	58	6	5.20	0.08	32

Table S3: Variants of SakSTAR 71–87 by Warmerdam et al., as well as by our dynamic programming algorithm, optimizing for ProPred (5% threshold) and allowing mutations according to BLOSUM, conservation, or FoldX. *E*: number of ProPred epitopes; *B*, *C*, and $\Delta\Delta G°$: total substitution penalty under BLOSUM, conservation, and FoldX, resp.; *S*: number of SMM-align epitopes.

Variant	SakSTAR 71–87	*E*	*B*	*C*	$\Delta\Delta G°$	*S*
wild type	`TAYKEFRVVELDPSAKI`	8	0	0.00	0.00	9
	**1 substitution**					
Warmerdam	`......A..........`	4	1	0.75	0.43	7
BLOSUM	`........T........`	2	0	-0.74	1.13	5
Conservation	`........D........`	2	1	0.72	1.90	3
FoldX	`........R........`	3	1	7.93	0.20	5
	**2 substitutions**					
Warmerdam	`......A..A.......`	8	2	2.48	1.00	11
BLOSUM	`........T....D...`	1	0	1.48	1.70	3
Conservation	`.....K..D........`	0	2	0.51	4.14	1
FoldX	`...A....R........`	1	2	15.94	0.10	4
	**3 substitutions**					
Warmerdam	`......A..A.A.....`	11	3	11.96	0.22	14
BLOSUM	`........T.M..D...`	0	0	9.50	1.41	3
Conservation	`n/a`	0	0	0	0	0
FoldX	`.......LK....P...`	1	2	3.09	-1.24	4
	**4 substitutions**					
Warmerdam	`...Q..S..S....S..`	9	2	25.37	2.12	7
BLOSUM	`n/a`	0	0	0	0	0
Conservation	`n/a`	0	0	0	0	0
FoldX	`...A..K.K....P...`	0	3	11.34	-0.58	3

Table S4: Variants of the full-length SakSTAR protein by Warmerdam et al., as well as by our dynamic programming algorithm, optimizing for ProPred (5% threshold) and allowing mutations according to BLOSUM, conservation, or FoldX. E: number of ProPred epitopes; B, C, and $\Delta\Delta G°$: total substitution penalty under BLOSUM, conservation, and FoldX, resp.; S: number of SMM-align epitopes.

Variant	SakSTAR 1–136	E	B	C	$\Delta\Delta G°$	S
wild type	SakSTAR	54	0	0.00	0.00	49
	**1 substitution**					
Warmerdam	R77A	50	1	0.75	0.43	47
BLOSUM	V112T	47	0	7.93	0.16	47
Conservation	M26D	41	1	1.15	1.89	41
FoldX	V112T	47	0	7.93	0.16	47
	**2 substitutions**					
Warmerdam	R77A,E80A	54	2	2.48	1.00	51
BLOSUM	V27T,V112T	40	0	15.82	2.24	46
Conservation	M26D,F125E	34	2	1.61	7.77	37
FoldX	G31D,V112T	41	1	8.76	0.12	44
	**3 substitutions**					
Warmerdam	R77A,E80A,D82A	59	3	11.96	0.22	55
BLOSUM	V27T,V79T,V112T	34	0	15.08	3.37	42
Conservation	M26D,V79T,F125E	28	2	0.86	8.90	33
FoldX	Y24H,G31D,V112T	36	2	9.72	0.19	40
	**4 substitutions**					
Warmerdam	K74Q,R77S,E80S,D82S	59	3	26.93	1.28	52
BLOSUM	V27T,V79T,V112T,K130E	29	0	15.84	4.80	40
Conservation	M26D,K50D,V79T,F125E	22	3	1.51	10.41	31
FoldX	Y24H,G31D,V79R,V112T	31	3	17.65	0.39	36
	**5 substitutions**					
BLOSUM	V27T,K50E,V79T,V112T,K130E	25	0	23.77	5.71	38
Conservation	M26D,I49D,V79T,V112K,F125E	16	4	0.27	11.83	27
FoldX	Y24H,G31D,Y62E,V79R,V112T	27	4	18.05	0.52	34
	**6 substitutions**					
BLOSUM	V27A,V29M,K50E,V79T,V112T,K130E	23	0	25.49	6.31	36
Conservation	M26D,I49D,Y62D,V79D,V112K,F125E	12	6	1.92	13.56	23
FoldX	Y24H,G31D,Y62E,V79R,V112T,V132W	24	5	25.61	0.54	32

Table S5: Variants of the full-length Epo protein by Tangri et al., as well as by our dynamic programming algorithm, optimizing for ProPred and allowing mutations according to BLOSUM, conservation, or FoldX. E: number of ProPred epitopes; B, C, and $\Delta\Delta G°$: total substitution penalty under BLOSUM, conservation, and FoldX, resp.; S: number of SMM-align epitopes.

Variant	Epo 1–166	E	B	C	$\Delta\Delta G°$	S
wild type	Epo	136	0	0.00	0.00	74
	**1 substitution**					
BLOSUM	R110Q	126	0	2.87	0.70	72
Conservation	R110G	128	1	2.81	1.87	75
FoldX	N147D	124	1	2.22	-0.33	70
	**2 substitutions**					
Tangri G2	L102P,S146D	118	1	4.97	6.06	65
Tangri G3	T107D,S146D	121	1	4.67	1.02	62
BLOSUM	R110Q,S146D	119	0	4.92	0.92	65
Conservation	R76A,R110G	120	2	5.48	3.47	76
FoldX	F48D,N147D	115	2	5.07	-0.59	65
	**3 substitutions**					
Tangri G4	L102G,T107D,S146D	113	2	7.59	6.09	57
Tangri G5	L102S,T107D,S146D	115	2	7.59	4.89	58
BLOSUM	R76K,V82T,R110Q	111	0	8.12	1.61	73
BLOSUM	R76Q,V82T,R110Q	111	0	8.91	1.78	72
Conservation	R76A,V82A,R110G	113	2	5.15	3.24	75
Conservation	V56E,R76A,R110G	113	3	7.95	3.29	69
FoldX	F48D,L93E,N147D	108	3	7.83	-0.55	62
FoldX	F48D,V82K,N147D	108	3	6.96	-1.18	63
	**4 substitutions**					
BLOSUM	R76K,V82T,R110Q,S146D	104	0	10.16	1.83	66
BLOSUM	R76Q,V82T,R110Q,S146D	104	0	10.96	2.00	65
Conservation	V56E,R76A,V82A,R110G	106	3	7.62	3.06	68
FoldX	F48D,V82E,L93E,N147D	101	4	10.01	-0.87	60
FoldX	F48D,V82K,L93E,N147D	101	4	9.71	-1.14	60
	**5 substitutions**					
BLOSUM	S71D,R76K,V82T,R110Q,S146D	98	0	12.91	2.47	62
BLOSUM	S71D,R76Q,V82T,R110Q,S146D	98	0	13.71	2.64	62
Conservation	V56E,R76A,V82A,R110G,R143Q	101	3	9.06	3.13	67
Conservation	V56E,R76A,V82A,L91G,R110G	101	4	10.40	4.77	66
FoldX	F48D,V82E,L93E,S146D,N147D	95	4	12.05	-0.65	57
FoldX	F48E,V82K,L93E,L130C,N147D	95	5	12.51	-0.90	58
FoldX	F48E,V82K,L93E,L130D,N147D	95	5	12.51	-0.94	57
FoldX	F48D,V82E,L93E,L130K,N147D	95	5	12.81	-1.00	57
FoldX	F48E,S71E,V82E,L93E,N147D	95	4	12.75	-1.02	58
FoldX	F48D,V82E,L93E,L130R,N147D	95	5	12.81	-1.55	57
FoldX	F48D,V82K,L93E,R131S,N147D	95	5	12.40	-0.92	60

Therapeutic Antibodies Case Study

Lazar et al. [1] introduced the concept of "human string content," or the percent identity between peptides derived from a test antibody sequence and corresponding peptides taken from a multiple sequence alignment of homologous human antibodies. They further developed algorithms to guide point mutations that optimize this metric for an antibody therapeutic's "humanness," and presumably its immunogenic potential. Importantly, an analysis of the structural implications of designed point mutations was not incorporated into the library planning strategy. Rather, this critical factor was applied as a downstream filter only after the libraries had been developed.

The approach was used to engineer deimmunized variants of the variable heavy and light chains from several murine antibody therapeutics. Ultimately, the variable domains would be grafted onto the corresponding human constant domains, nullifying the need to make substitutions in the constant domains of the heavy and light chains. This antibody humanization via human string content strategy was implemented in a three-stage, iterative process that entailed construction of small point mutant libraries, functional screening of library members to isolate the most promising candidates, and use of the isolated sequences as templates in construction of the next generation library. Ultimately, only two of the four murine antibodies were pursued to a fully humanized endpoint. The humanized AC10 variant possessed 42 point mutations relative to the murine template, and the humanized 225 variant possessed 53 compared to its murine starting point. Both humanized antibodies exhibited biological activity equivalent to or exceeding that of their murine counterparts, and of course both engineered antibodies possessed improved human string content. The actual immunogenicity of the humanized sequences was not experimentally evaluated.

Unfortunately, the authors do not provide sequence information for the engineered antibodies, precluding a direct comparison of our results with their own. However, we obtained the wild-type sequences for three proteins: the anti-Her2/neu antibody 4D5, the anti-EGFR antibody 225, and the anti-EpCAM antibody 17-1A. To find a family of related mammalian antibodies, we PSI-BLASTed each sequence using the EBI server (http://www.ebi.ac.uk/Tools/psiblast/) with default parameters including UniProt Knowledgebase, BLOSUM-62, and a 0.001 threshold. Iterations were repeated until no new sequences were added to the alignment, and sequences under 35% identity to the original were discarded, leaving 500 sequences. We then ran our dynamic programming algorithm on these sequences using the conservation metric, allowing up to 16 substitutions ($\approx$ 15% of the residues).

Fig. S2 shows the epitope score profiles for a 16-substitution conservation-based variant (see Supplementary Material for corresponding figures for other antibodies, which have similar characteristics). This figure illustrates a significant property of our approach: it preferentially targets mutations to both promiscuous epitopes (i.e., an epitope that binds to more than one MHC II allele) and to promiscuous residues (i.e., an amino acid that is a key element of more than one immunogenic peptide).

Variants of the 4D5 antibody heavy and light chains with 1, 2, 4, 8, and 16 substitutions each are detailed in Table S6. These variants did not take into account the need to avoid mutation of key binding residues. A practical experimental plan would require masking of these residues in order to restrict mutations from critical areas of the complementary determining regions. However, even in the absence of this masking procedure, the efficiency of our deimmunization planning is evident. At the 16-substitution level, we are able to reduce epitope score by about 70-90%; this compares favorably to the published studies, which required more than four times that many substitutions. Furthermore, the conservation scores show that the substitutions employed are fairly conservative, even compared to our 5% weighted threshold which give a penalty of about 3 per mutation. (Negative scores result when a substituted residue is more represented in the family than is the wild-type residue.) The progression of epitope and conservation score further allows easy expression of a cost-benefit analysis: how many eliminated epitopes does each mutation buy, and at what risk to stability/activity by differentiating from the family?

4D5

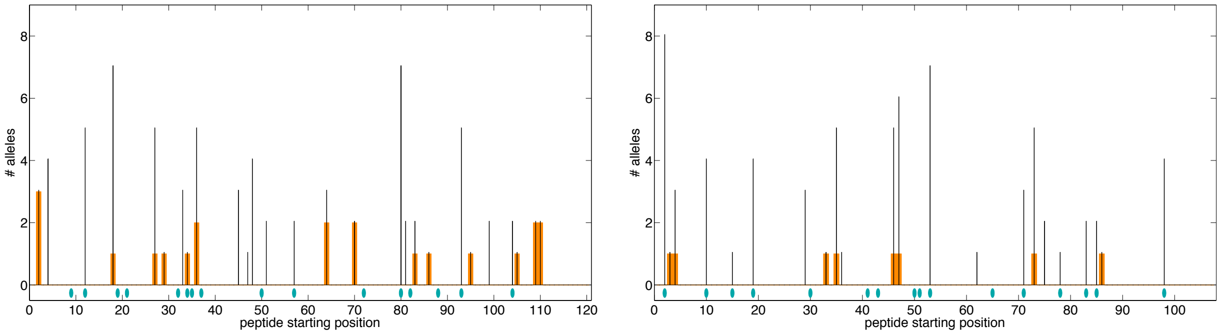

225

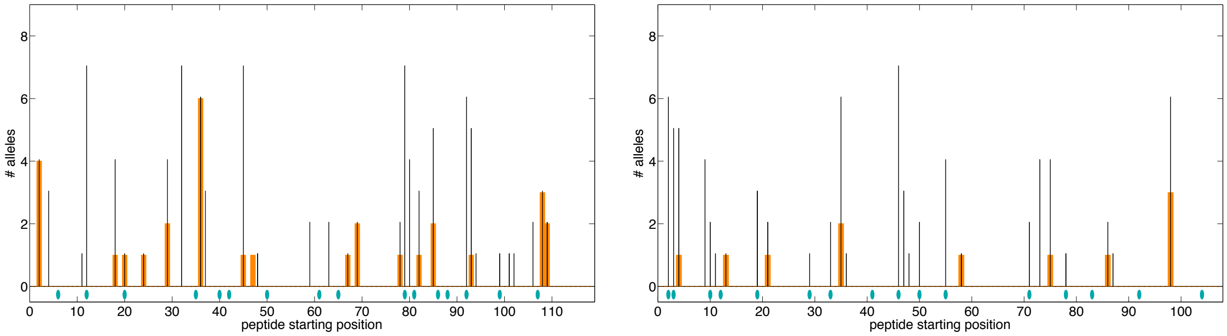

17-1A

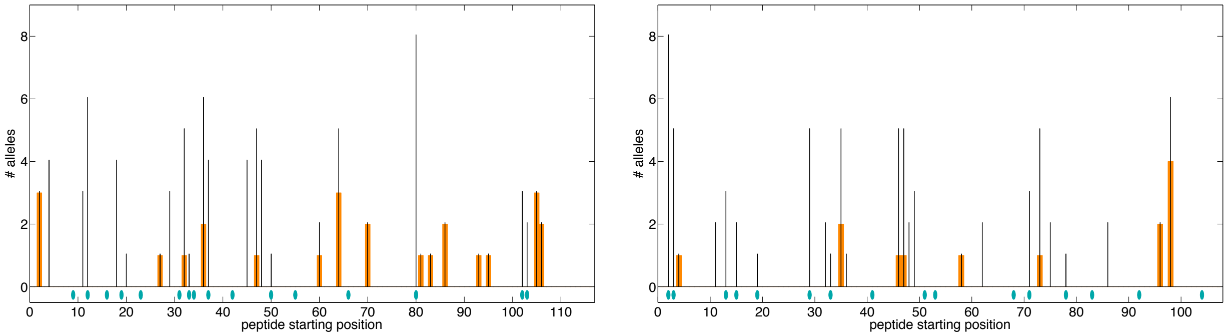

Figure S2: Antibody immunogenicity profiles in wild-types vs. optimal 16-mutation variant. x-axis: starting position of each 9mer; y-axis: predicted number of alleles recognizing the 9mer. Thin black bars indicate wild-type scores and thick orange bars indicate variant scores. Note: wild-type epitope scores are always greater than or equal to corresponding variant ones; i.e., we never introduce new epitopes. Blue ellipses indicate mutated positions (refer to Table S7 and Table S8). Left: variable heavy chain; Right: variable light chain.

Table S6: Variants of the 4D5 antibody by our dynamic programming algorithm, optimizing for ProPred and allowing mutations according to conservation. E: number of ProPred epitopes; C: total conservation penalty; S: number of SMM-align epitopes.

Variant	Substitutions	E	C	S
	**4D5 VH**			
wt		78	0.00	57
1	R50E	70	0.58	54
2	V12K,R50E	63	2.32	52
4	V12K,R50E,Y80S,W99D	51	2.78	49
8	V12K,K19S,V37I,R50E,Y57D,Y80S,V93T,F104D	36	8.88	42
16	P9G,V12K,K19S,S21T,T32Y,I34W,H35G,V37I, R50E,Y57D,A72R,Y80S,Q82E,S88A,V93T,F104D	21	10.70	37
	**4D5 VL**			
wt		70	0.00	43
1	F53D	60	-2.55	38
2	I2S,F53D	52	-1.23	34
4	I2S,F10S,F53D,L83E	41	-5.18	33
8	I2S,F10S,V19A,L46T,F53D,V78M,L83E,F98G	24	1.37	32
16	I2S,F10S,V15P,V19A,N30S,G41D,S43P,S50G, A51D,F53D,N65S,F71A,V78M,L83E,V85D,F98G	8	-5.39	25

Table S7: Variants of the 225 antibody by our dynamic programming algorithm, optimizing for ProPred and allowing mutations according to conservation. E: number of ProPred epitopes; C: total conservation penalty; S: number of SMM-align epitopes.

Variant	Substitutions	E	C	S
	**225 VH**			
wt		99	0.00	60
1	V12K	90	2.45	59
2	V12K,A98D	82	1.08	58
4	V12K,V50E,F79S,A98D	68	1.37	50
8	V12K,H35G,S40P,V50E,F79S,N88D,A98D,A107D	51	-2.89	44
16	Q6E,V12K,I20V,H35G,S40P,G42E,V50E,T61D, S65D,F79S,K81S,Q86T,N88D,I92T,L99D,A107D	30	-3.75	44
	**225 VL**			
wt		77	0.00	41
1	I10T	68	-0.65	40
2	L3A,I10S	61	-3.63	36
4	L3A,I10S,L46T,I55E	47	-3.31	26
8	L3A,I10S,V19A,N41D,L46T,I55E,V78L,I83E	30	-6.74	23
16	I2S,L3Q,I10S,S12P,V19A,I29D,I33A,N41D, L46T,Y50G,I55P,F71A,V78L,I83E,N92D,L104V	11	-3.55	18

Table S8: Variants of the 17-1a antibody by our dynamic programming algorithm, optimizing for ProPred and allowing mutations according to conservation. E: number of ProPred epitopes; C: total conservation penalty; S: number of SMM-align epitopes.

Variant	Substitutions	E	C	S
	**17-1A VH**			
wt		88	0.00	53
1	V50E	77	0.41	51
2	V12K,V50E	68	2.11	50
4	V12K,I34W,V50E,Y80S	54	6.00	49
8	V12K,I34W,V37I,G42E,V50E,G66D,Y80S,W102G	41	10.96	42
16	A9G,V12K,T16E,K19S,K23A,N31D,L33W,I34M, V37I,G42E,V50E,S55D,G66D,Y80S,W102G,F103G	25	4.89	36
	**17-1A VL**			
wt		75	0.00	43
1	I2S	67	1.34	41
2	I2S,A51D	60	2.47	37
4	I2S,V3Q,A51D,N53D	49	4.80	35
8	I2S,V3Q,M13A,V29D,E41D,A51D,N53D,Q79E	32	2.87	25
16	I2S,V3Q,M13A,V15P,V19A,V29N,V33A,E41D, A51D,N53D,A68G,F71A,V78M,L83E,Y92D,L104V	13	0.88	29

References

[1] Lazar GA, Desjarlais JR, Jacinto J, Karki S, Hammond PW: **A molecular immunology approach to antibody humanization and functional optimization**. *Mol. Immunol.* 2007, **44**:1986–1998.
